# From brain to education through machine learning: Predicting literacy and numeracy skills from neuroimaging data

**DOI:** 10.1162/imag_a_00219

**Published:** 2024-07-03

**Authors:** Tomoya Nakai, Coumarane Tirou, Jérôme Prado

**Affiliations:** Lyon Neuroscience Research Center (CRNL), INSERM U1028 - CNRS UMR5292, University of Lyon, Bron, France; Araya Inc., Tokyo, Japan

**Keywords:** education, development, neuroscience, machine learning, learning disability

## Abstract

The potential of using neural data to predict academic outcomes has always been at the heart of educational neuroscience, an emerging field at the crossroad of psychology, neuroscience, and education sciences. Although this prospect has long been elusive, the exponential use of advanced techniques in machine learning in neuroimaging may change this state of affairs. Here we provide a review of neuroimaging studies that have used machine learning to predict literacy and numeracy outcomes in adults and children, in both the context of learning disability and typical performance. We notably review the cross-sectional and longitudinal designs used in such studies, and describe how they can be coupled with regression and classification approaches. Our review highlights the promise of these methods for predicting literacy and numeracy outcomes, as well as their difficulties. However, we also found a large variability in terms of algorithms and underlying brain circuits across studies, and a relative lack of studies investigating longitudinal prediction of outcomes in young children before the onset of formal education. We argue that the field needs a standardization of methods, as well as a greater use of accessible and portable neuroimaging methods that have more applicability potential than lab-based neuroimaging techniques.

## 1 Introduction

The past few decades have seen a rapid increase in our understanding of how the brain changes over development and learning, leading a number of neuroscientists to consider implications of these findings for education. This has led to the emergence of the field of educational neuroscience (Ansari & Coch, 2006;Goswami, 2004,^2006^), defined in a recent review (Thomas et al., 2019) as “an interdisciplinary research field that seeks to translate research findings on neural mechanisms of learning to educational practice and policy.” However, this general endeavor has not been unchallenged. Critics have notably claimed that neuroscience findings are too remote from the classroom to be informative and to have practical implications for children or educational systems (Bruer, 1997). Others have argued that behavioral measures are more practical to characterize children’s cognitive capacities than neuroimaging measures (Bowers, 2016).

In an earlier review,Gabrieli et al. (2015)argued otherwise and suggested that brain measures obtained through neuroimaging techniques may be useful for predicting future academic outcomes and, therefore, help design interventions, as well as for evaluating the success of interventions. A relatively limited number of studies were available at the time of Gabrieli et al.’s review. However, significant progress has since been made in both neuroimaging and machine-learning techniques. The term “machine learning” refers here to a set of computational methods that involve the development of algorithms and statistical models relying on patterns and inference derived from data. These computational methods typically use past information to improve their performance or to make accurate predictions over time (Mohri et al., 2012). Because these technological advances are changing the landscape of what may be possible in terms of the prediction of outcomes from neural signals, we aimed here to provide an updated review of recent advances in neuroscience and machine learning that may have application to both education and the treatment of neurodevelopmental disorders. Though the present review primarily focuses on the methodological framework, challenges, and main findings from these studies, we will also end by discussing the potential practical applications of this line of research.

The present review largely focuses on findings in the domains of literacy and numeracy skills (and associated disorders) for two reasons. First, literacy and numeracy skills are considered fundamental to modern science and technologies, and difficulties in acquiring these abilities may negatively impact academic attainment and financial well-being (Estrada-Mejia et al., 2016). Predicting reading and mathematical difficulties in children has, therefore, critical societal relevance. Second, literacy and numeracy are probably the academic domains for which the most progress has been made in developmental cognitive neuroscience over the past decades. We will, however, also include in our review several studies that have focused on other cognitive factors relevant to education. Finally, we will highlight future directions for studies aiming to apply machine learning to neural data in order to predict and improve educational outcomes.

## 2 Predicting Educational Outcomes From Brain Activity: Methodological Considerations

Gabrieli et al. (2015)pointed out that the term “prediction” can have at least three different meanings in studies. In its weakest form, the term might be used to describe a correlation between two sets of variables obtained at the same time point. In a slightly stronger form, it can also be used to describe a correlation between two sets of variables obtained at different time points. In its strongest form, “prediction” may describe a model generalization to out-of-sample individuals, which typically relies on machine learning. This third meaning is arguably the closest to the definition of a “prediction” in common language. Studies demonstrating an out-of-sample generalization have also the most practical relevance because they suggest that a model would be applicable to novel data that are not specific to a given sample.

The present review exclusively focuses on the term “prediction” as describing generalization to out-of-sample individuals, and, therefore, only includes studies demonstrating such generalization. As a side note, not all neuroimaging studies using machine-learning techniques are relevant to the question of individual differences in academic performance, learning, or development. For instance, studies may use machine learning to test differences in spatial distributions of neural activity across tasks (Nakai et al., 2023). These studies are not included in the present review either.

**Fig. 1.**
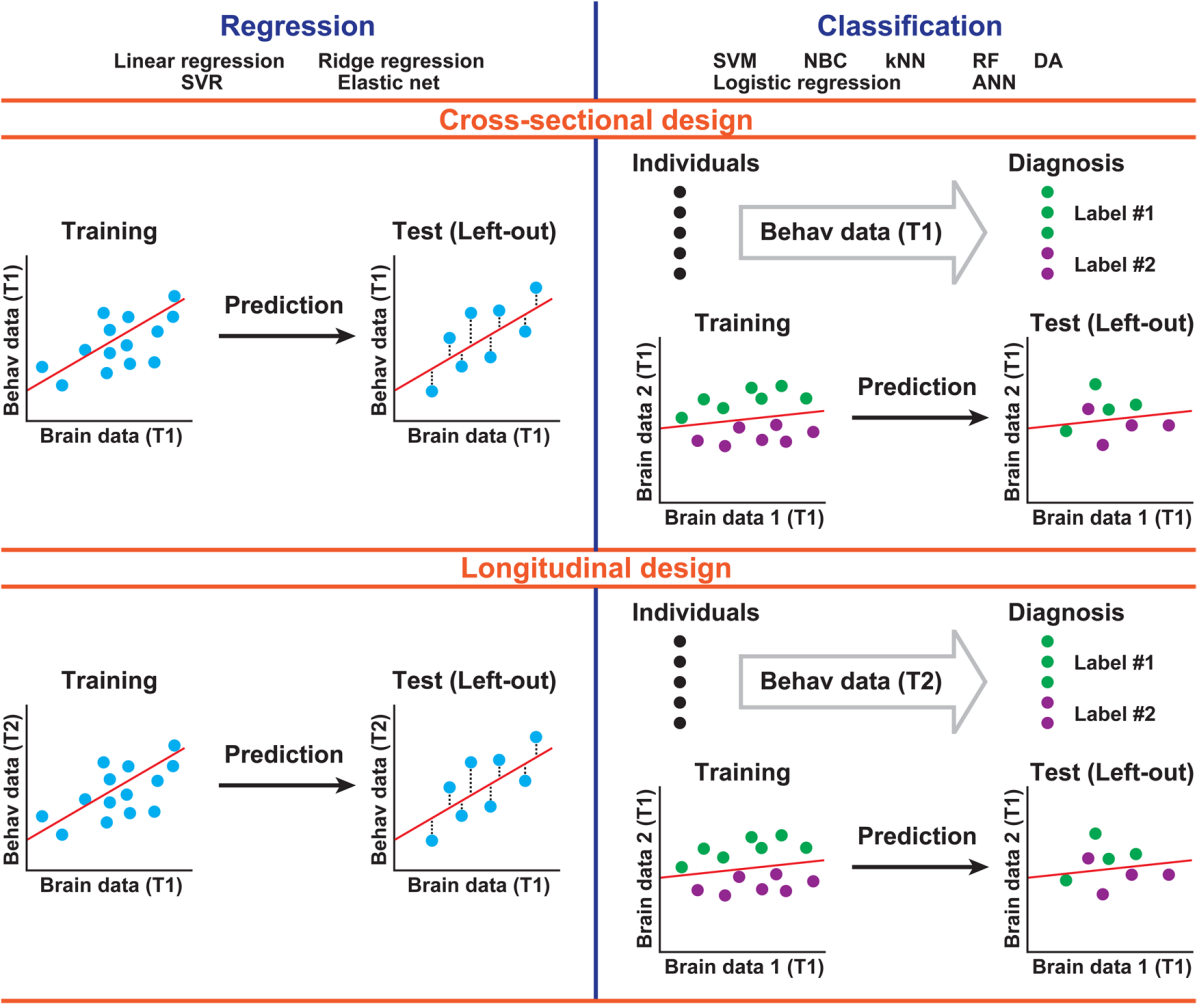
Schematic chart outlining the methodology used in neuroimaging studies reviewed here. Studies can be categorized into following a cross-sectional or a longitudinal design (rows), as well as a regression or a classification approach (columns). T1, time point 1; T2, time point 2. Note that although cross-sectional design can be applied to multiple time points, we only describe the case of T1 to avoid confusion with the longitudinal design. Furthermore, we also simplified the description of the longitudinal design by excluding cases of using differences of behavioral data (T2 - T1) as explained variables. Labels #1 and #2 indicate discrete categorization of individuals (e.g., typically developing or learning disabled). ANN, artificial neural network; DA, discriminant analysis; kNN, k-nearest neighbors; NBC, naïve Bayes classifiers; RF, random forest; SVM, support vector machine; SVR, support vector regression.

Broadly speaking, previous neuroimaging studies using machine learning to predict educational outcomes can be divided into two categories. The first category (Fig. 1, top row) encompasses studies using a cross-sectional design, such that different participants are evaluated at one (T1) or several time points (T1 and T2). The second category (Fig. 1, bottom row) includes studies using a longitudinal design, such that the same participants are evaluated at different time points (T1 and T2). These time points can be separated by days, weeks, or even years. Note that cross-sectional and longitudinal studies may use supervised learning to predict either a continuous distribution of achievement (e.g., reading, math) scores from brain activity or discrete categorical labels such as presence or absence of learning disability. While the former relies on regression analyses (Fig. 1, left column), the latter involves classification analyses (Fig. 1, right column) (Bishop, 2006).

Note that the three typical meanings of “prediction” inGabrieli et al. (2015)can be categorized along the dimensions of “in-sample correlation vs. out-of-sample prediction” and “cross-sectional vs. longitudinal.” That is, the first two meanings are similar in that they both focus on in-sample correlation but are different because one uses a cross-sectional design and the other a longitudinal design. The third meaning (out-of-sample prediction) can also be applied to both cross-sectional and longitudinal data (Fig. 1). In both cases, machine-learning models are trained with a subset of samples, and their generalizability is tested with left-out samples.

Regression and classification analyses use different analytic strategies. For instance, regression analysis as it is applied to a cross-sectional design (Fig. 1, upper left cell) relies on the generation of a predictive model based on the relationship between brain and behavioral data across participants from the training set at T1. The trained model is then used to predict behavior from brain data in left-out participants, also at T1. Regression analysis as it is applied to a longitudinal design (Fig. 1, bottom left cell) relies on the generation of a predictive model based on the relationship between brain data at T1 and behavioral data at T2 across participants from the training set. The trained model is then used to predict behavior at T2 from brain data at T1 in left-out participants. Classification analysis as it is applied to a cross-sectional design (Fig. 1, upper right cell) relies on an association between a discrete categorization of participants from the training set according to behavioral labels defined at T1 and their brain data at T1. This trained model is then used to assign labels to left-out participants based on their specific brain data, also at T1. Classification analysis as it is applied to a longitudinal design (Fig. 1, bottom right cell) relies on an association between a discrete categorization of participants from the training set according to behavioral labels defined at T2 (e.g., typically developing or learning disabled) and their brain data at T1. This trained model is then used to assign labels to left-out participants based on their specific brain data at T1. The specific methodologies underlying these analyses are discussed in a later section (see Studies Use a Range of Machine-Learning Methods). The present study does not include data from human or animal subjects and does not require approval from the ethics committee or informed consent.

## 3 Can Neuroimaging Studies Predict Literacy Skills?

A number of cross-sectional (Table 1) and longitudinal (Table 2) neuroimaging studies have attempted to use brain data to predict literacy skills (seeSupplementary Informationfor the selection criteria of articles and the methodology used to generate the tables). For example, using regression in a cross-sectional design,He et al. (2013)showed that gray matter (GM) structural MRI (sMRI) data from adult participants could predict various language abilities (phonological decoding, form-sound association, and naming speed) decomposed from a set of behavioral measures.Xu et al. (2015)further used fractional amplitude of low-frequency fluctuations (ALFF) in resting fMRI (rest-fMRI) data to predict reading test scores (efficiency of mapping orthography to semantic) of adult participants. Subsequent studies have focused on large datasets of adult participants provided by the Human-Connectome Project (HCP) (Van Essen et al., 2013). These studies used either the Oral Reading Recognition Test and/or Picture Vocabulary Test combined with different types of brain data: sMRI (Cui et al., 2018;Kristanto et al., 2020), functional connectivity (FC) of rest-fMRI (Kristanto et al., 2020;Yuan et al., 2023), diffusion MRI (dMRI) (Kristanto et al., 2020), and task-fMRI (language, working memory, and motor tasks) (Tomasi & Volkow, 2020). Together, these studies show that it is possible to predict individual differences in literacy skills with different sources of neuroimaging data, indicating that such skills are related to brain data over multiple dimensions.

**Table 1.** Cross-sectional prediction studies for literacy.

Study	Target ability/groups	Sample size	Mean age/age range	Data type	Technique	Cross-validation	Max prediction accuracy	Brain areas	Selection method of brain areas
He et al. (2013)	Phonological decoding, form-sound association, naming speed	253	21.5	sMRI (GM)	Linear SVR	10-fold CV	Phonological decoding, *r* = 0.26; form-sound association, *r* = 0.23; naming speed, *r* = 0.24	Phonological decoding, 4 regions including L. SPL and precuneus; form-sound association, 9 regions in the temporal cortex and hippocampus; naming speed, 11 regions in the frontal, temporal, and parietal cortices	Searchlight
Mossbridge et al. (2013)	Subjects with good or poor reading scores	28	18-29	Task-EEG (sentence comprehension)	RF	Repeated selection of 35% of subjects (1000 times)	88.3%	Medial frontal channel	Weight values
Xu et al. (2015)	Reading scores	263	22.1	rest-fMRI (ALFF)	Linear regression	4-fold CV	*r* = 0.24	Bilateral PreCG, STG	Predetermined ROIs. Nonindependent
Bailey et al. (2016)	Dyslexia, SRCD	41 (14 dyslexia, 11 SRCD, 16 TD)	Dyslexia, 12.5; SRCD, 11.5; TD, 11.9	sMRI (GM)	Linear SVM	LOOCV	92.5% (SRCD vs. TD)	Large portions of the frontal, temporal, parietal, occipital cortices, subcortex and cerebellum	Weight values
Cui et al. (2016)	Dyslexia	61 (28 dyslexia, 33 TD)	Dyslexia, 11.6; TD, 11.8	sMRI (WM), dMRI (FA, mean, axial, radial diffusivity)	Linear SVM, Logistic regression	LOOCV	83.6% (SVM)	43 (SVM) and 40 (Logistic regression) connections across the brain	CV within training data
Tamboer et al. (2016)	Dyslexia	First sample: 49 (22 dyslexia, 27 TD); second sample: 876 (60 dyslexia, 816 TD)	First sample, dyslexia, 20.7; TD, 20.3; second sample, dyslexia, 22.5; TD, 22.9	sMRI (GM)	Linear SVM	LOOCV	First sample, 80.0%; Second sample, 59.0%	L. IPL, bilateral FG	Predetermined ROIs. Independent
Zare et al. (2016)	Familial risk of dyslexia	24 (12 with familial risk, 12 without risk)	0.5	rest-EEG (FC)	SVM (linear and 3 nonlinear kernels)	LOOCV	79.2% (linear and cubic)	Left frontal and bilateral parietal channels	Predetermined channels. Nonindependent
Płoński et al. (2017)	Dyslexia	236 (130 dyslexia, 106 TD)	8.5-13.7	sMRI (volume, cortical thickness, surface area, folding index, and mean curvature)	Linear SVM, Logistic regression, RF	LOOCV and repeated 10-fold CV (100 times)	65.0%	L. MTG, L. STG, L. frontal pole, L. precuneus	CV within training data
Cui et al. (2018)	Reading scores, Dyslexia	First sample, 507; second sample, 372; third sample, 67 (25 dyslexia, 42 TD)	First sample, 22-35; second sample, 22-35; third sample, 11.0	sMRI (GM)	Elastic net	3-fold CV	First sample, *r* = 0.43; second sample, *r* = 0.34; third sample, *r* = 0.24	Large portions of the frontal, temporal, parietal, occipital cortices, subcortex and cerebellum	Weight values
Dimitriadis et al. (2018)	Dyslexia	52 (25 dyslexia, 27 TD)	Dyslexia, 12.2; TD, 11.4	rest-MEG	kNN, SVM	Repeated 5-fold CV (100 times)	97.0%	Parietal and temporal channels	Correlation between weight values and behavioral scores
Zainuddin et al. (2018)	Dyslexia	33 (17 poor dyslexia, 8 capable dyslexia, 8 TD)	7-12	task-EEG (word comprehension)	Nonlinear SVM	10-fold CV	91.0%	No specific information	N.A.
Barranco-Gutiérrez (2020)	L2 speakers and natives	19 L2 English speakers and 25 natives	L2 speakers, 31.9; natives 28.2	dMRI	ANN	75% for training, 10% for validation, 15% for testing	97.0%	Corpus callosum	Predetermined ROIs. Independent
Cignetti et al. (2020)	Dyslexia, DCD	136 (45 dyslexia, 20 DCD, 29 comorbid, 42 TD)	Dyslexia, 10.2; DCD, 10.0; comorbid, 10.2; TD, 10.1	rest-fMRI (FC)	Linear SVM	LOOCV	75.9% (comorbid vs. TD)	Default mode, dorsal attention, ventral attention, frontoparietal networks	Weight values
Kristanto et al. (2020)	Reading scores	998	22-35	sMRI (thickness, myelination, sulcus depth), rest-fMRI (FC), dMRI (connectivity strength)	Linear regression	2-fold CV with LOOCV in each fold	*r* = 0.21	Large portions of the frontal, temporal, and parietal cortices	CV within training data
Lei et al. (2020)	Second language proficiency	40 native Japanese (20 high and 20 low proficiency), 38 native English speakers (19 high and 19 low proficiency)	Japanese, high, 28.1; low, 29.4; English, high, 29.3; low, 28.5	task-fNIRS	Linear SVM, kNN, Sparse logistic regression	LOOCV	81.9% (English, SVM)	L. MFG, L. PreCG, L. ITG, L. PostCG, L. AG, bilateral STG, bilateral MTG	Sparse canonical correlation analysis
Tomasi and Volkow (2020)	Reading and vocabulary scores	424	29.0	rest-fMRI (FC), task-fMRI (language, working memory, and motor tasks, FC)	Linear regression	2-fold CV with LOOCV in each fold	*R* = 0.33	Frontoparietal and default mode networks	CV within training data
Zahia et al. (2020)	Dyslexia	55 (19 dyslexia, 17 monocular vision, 19 TD)	Dyslexia, 10.5; monocular vision, 10.4; TD, 10.0	task-fMRI (lexical decision, orthographic matching, semantic categorization)	ANN (3D CNN)	4-fold CV	72.3%	Bilateral IFG, MTG, STG, precuneus, FG, L. AG, L. medial temporal	Predetermined ROIs. Independent
Formoso et al. (2021)	Dyslexia	48 (16 dyslexia, 32 TD)	Dyslexia, 8.0; TD, 7.8	task-EEG (auditory stimuli listening)	NBC	5-fold CV	90.0% (Beta, 16 Hz)	Alpha, beta, delta, theta, gamma bands	N.A.
Mascheretti et al. (2021)	Dyslexia	44 (22 dyslexia, 22 TD)	Dyslexia, 14.1; TD, 13.2	task-fMRI (visual detection)	Multiple kernel learning SVM	10-fold CV	65.9%	11 ROIs including R. SPL, L. IPL, R. IFG, and occipital cortex	Weight values
McNorgan (2021)	High-skilled and poor readers	First sample, 28 (14 high-skilled and 14 poor readers); second sample, 10 (5 high-skilled and 5 poor readers)	First sample, 8-13; second sample, 8-14	task-fMRI (rhyme judgment, multiplication, FC)	ANN (MLP)	10-fold CV, 3-fold CV	First sample, 94.0%; second sample, 96.0% (of functional connectivity)	115 ROIs across the brain	Predetermined ROIs. Nonindependent
Tomaz Da Silva et al. (2021)	Dyslexia	32 (16 dyslexia, 16 TD)	Dyslexia, 9.6; TD, 8.4	task-fMRI (word reading)	four ANNs (grammar-based genetic programming [GGP] CNN, GGP 3D CNN, LeNet-5, LeNet-5 3D), linear SVM	80% for training, 10% for validation, 10% for testing	94.8% (GGP 2D CCN)	Large portions of the frontal, parietal, temporal, and occipital cortices	Weight values
Usman et al. (2021)	Dyslexia	Dyslexia, 91; TD, 57	Dyslexia, 11.4; TD, 19-30	sMRI (GM), task-fMRI (rhyming, spelling, semantic decision), rest-fMRI, dMRI	ANN (two-ways cascaded CNN, ResNet-50, Inception V3)	Repeated 10-fold CV (10 times)	94.7% (ResNet50)	L. STG, L. OTG, lateral cerebellum	Predetermined ROIs. Independent
Yu et al. (2022)	Familial risk of dyslexia	98 (35 with familial risk, 63 without risk)	Risk, 8.9; without risk, 8.3	rest-fMRI (FC)	Linear SVM	LPOCV	55.0%	L. FG	Predetermined ROIs. Nonindependent. Weight values
Joshi et al. (2023)	Dyslexia	192 (96 dyslexia, 96 TD)	Dyslexia, 9.9; TD, 9.8	sMRI (GM, WM)	ANN (autoencoder), SVM, RF	Repeated sampling (100 times) with 80% for training, 20% for testing	75.0% (ANN)	L. IPL, R. orbitofrontal, L. STG	Classification accuracy with image perturbation
Nemmi et al. (2023)	Dyslexia, DCD	136 (45 dyslexia, 20 DCD, 29 comorbid, 42 TD)	Dyslexia, 10.2; DCD, 1.0; comorbid, 10.2; TD, 10.1	sMRI (GM, WM), rest-fMRI (ALFF, local and global correlation)	RF, linear SVM	Repeated 10-fold CV (10 times)	Dyslexia, 79.0%; DCD, 58.0%; comorbid, 62.0% (SVM)	12 ROIs including L. cerebellum, R. MFG, R. SFG, R. LOC, L. insula, R. putamen, R. insula, and R. STG	Predetermined ROIs. Nonindependent
Yuan et al. (2023)	Reading and vocabulary scores	522	28.5	rest-fMRI (FC)	Relevance vector regression	LOOCV	Reading test, *r* = 0.25; vocabulary test, *r* = 0.29	4 networks across frontal and temporal cortices	Predetermined ROIs. Independent
Zhang et al. (2023)	Second language proficiency	47 (15 low-, 16 moderate-, 16 high-proficiency)	22.7	task-fMRI (story listening)	SVM, Ridge regression	LOOCV	49.0%, *r* = 0.47	Large portions of the frontal, parietal, temporal, and occipital cortices	Predetermined ROIs. Nonindependent

DCD, developmental coordination disorder; SRCD, specific reading comprehension deficit; TD, typically developing; dMRI, diffusion magnetic resonance imaging; fMRI, functional MRI; sMRI, structural MRI; EEG, electroencephalography; MEG, magnetoencephalography; fNIRS, functional near-infrared spectroscopy; GM, gray matter; WM, white matter; ALFF, amplitude of low-frequency fluctuations; FC, functional connectivity; LOOCV, leave-one-out cross-validation; LPOCV, leave-pair-out cross-validation; ROI, region-of-interest; AG, angular gyrus; FG, fusiform gyrus; IFG, inferior frontal gyrus; IPL, inferior parietal lobule; ITG, inferior temporal gyrus; LOC, lateral occipital cortex; MFG, middle frontal gyrus; MTG, middle temporal gyrus; PreCG, precentral gyrus; PostCG, postcentral gyrus; SFG, superior frontal gyrus; SMG, supramarginal gyrus; SPL, superior parietal lobule; STG, superior temporal gyrus.

**Table 2.** Longitudinal prediction studies for literacy.

Study	Target ability/groups	Sample size	Mean age/age range	Data type	Technique	Cross-validation	Max prediction accuracy	Brain areas	Selection method of brain areas
Hoeft et al. (2007)	Reading scores after one school year	64	T1: 10.0, T2: 10.6	task-fMRI (rhyme judgment), sMRI (GM, WM)	Multiple linear regression	LOOCV	Unclear	R. FG, L. MTG, R. MFG, L. STG, L. IPL	Predetermined ROIs. Nonindependent
Hoeft et al. (2011)	Improvement of reading scores in dyslexia after 2.5 years	25 (12 dyslexia with gain, 13 without gain)	T1: with gain, 14.5; without gain, 14.6; T2: with gain, 17.0; without gain, 16.0	task-fMRI (rhyme judgment), dMRI (FA)	Linear SVM	LOOCV	92.0%	Whole brain, R. IFG, R. SLF	Predetermined ROIs. Independent
Bach et al. (2013)	Reading scores after 2 years	19	T1: 6.4, T2: 8.4	task-EEG, task-fMRI (word comprehension)	DA	LOOCV	94.1%	L. FG, L. occipitotemporal channels	Predetermined ROIs. Independent
Skeide et al. (2016)	Dyslexia after 1.7 years and at the end of the first grade	First sample: 34 (17 dyslexia, 17 TD); second sample 20 (10 dyslexia, 10 TD)	T1: First sample, dyslexia, 10.4; TD, 10.6; second sample, dyslexia, 5.6; TD, 5.8; T2: unclear	sMRI (GM, WM)	Linear SVM	10-fold CV	First sample: 73.5%; second sample: 75.0%	L. FG	Prediction accuracy, predetermined ROIs. Nonindependent
Yu et al. (2020)	Familial risk of dyslexia	81 (35 with risk, 34 without risk, 12 dyslexia and with familial risk)	T1: with risk, 5.5; without risk, 5.4; dyslexia, 5.8; T2: with risk, 8.7; without risk, 9.0; dyslexia, 8.3	task-fMRI (phonological processing)	Linear SVM	15-fold CV	68.3%	R. IFG, L. AG	Searchlight
Feng et al. (2021)	Learning outcomes after 7 days training in an artificial language	33	T1/T2: 22.3	task-fMRI (vocabulary and grammar training)	Least-squares SVR	Repeated 10-fold CV (10000 times)	*r* = 0.61	23 ROIs across frontoparietal, perisylvian, salience, and default mode networks	Weight values
Beyer et al. (2022)	Literacy skill after 2 years	42	T1: 5.6, T2: 8.3	sMRI (GM, surface area, local gyrification)	Elastic net	LOOCV, repeated 10-fold CV (50 times)	*r* = 0.80	L. IFG, STG, MTG, insula, ITG, FG, SMG, AG	Predetermined ROIs. Independent. Weight values

SLF, superior longitudinal fasciculus.

Other studies have attempted to use neuroimaging data to classify between participants with and without dyslexia, a specific learning difficulty in word recognition, word decoding, and spelling abilities, with otherwise normal intelligence (American Psychiatric Association, 2013). For example,Tamboer et al. (2016)classified adults with and without dyslexia using sMRI (GM) data.Cui et al. (2016)andJoshi et al. (2023)further showed that such classification was not limited to adults based on dMRI and sMRI data, respectively. Using sMRI (GM) data, but with a larger sample size including children from three different countries (130 children with dyslexia and 106 typically developing children),Płoński et al. (2017)replicated successful dyslexia classification. Finally, some studies have reported successful classification between children with and without dyslexia based on task-electroencephalography (EEG) with word comprehension (Zainuddin et al., 2018) and auditory stimuli listening (Formoso et al., 2021), and resting magnetoencephalography (MEG) signals (Dimitriadis et al., 2018). Although many of the studies above rely on rest-fMRI or sMRI data, more recent studies have also used task-fMRI data. For example,Mascheretti et al. (2021)classified dyslexic from nondyslexic children using a visual detection task, whereasTomaz Da Silva et al. (2021)used a word-reading task. Finally,Zahia et al. (2020)used three different reading tasks to classify children with dyslexia, monocular vision (due to ocular motility disorders), and control groups.

Studies have also attempted to distinguish between different subtypes of language-related disorders and language proficiency levels.Bailey et al. (2016)were able to distinguish children with dyslexia from those with specific reading comprehension deficits (SRCDs) based on their sMRI (GM) data. SRCD differs from dyslexia in that affected children have difficulty in reading comprehension despite adequate phonemic decoding (Landi & Ryherd, 2017).Cignetti et al. (2020)andNemmi et al. (2023)classified between children with dyslexia and with developmental coordination disorder (DCD) using rest-fMRI and sMRI (GM and white matter [WM]) data.Zare et al. (2016)andYu et al. (2022)classified whether children’s families had a history of dyslexia using rest-EEG and rest-fMRI data, respectively. One study has also used functional near-infrared spectroscopy (fNIRS) study to classify between higher and lower second language proficiency groups (Lei et al., 2020).Barranco-Gutiérrez (2020)classified between adults who are native English speakers and those who learned English as a second language.Zhang et al. (2023)classified second language (English) proficiency levels (high, moderate, low) of Chinese speakers and further predicted listening comprehension scores using fMRI with a story listening task.Mossbridge et al. (2013)found that good and poor readers were separable using EEG data during a sentence comprehension task.

In comparison with the number of studies that have used cross-sectional designs to predict literacy outcomes, a much smaller number of studies have used longitudinal designs to make out-of-sample predictions of literacy outcomes (Table 2). A pioneering study byHoeft et al. (2007)combined both task-fMRI (rhyme judgment) and sMRI (GM and WM) data as inputs of multiple linear regression models. The authors found that brain data could predict later reading scores at the end of the same year.Bach et al. (2013)combined task-EEG and task-fMRI data (word comprehension) to predict reading scores measured 2 years later. InFeng et al. (2021), subjects underwent grammar training of an artificial language. Their final learning outcomes were predicted from task-fMRI data during training in earlier sessions.Beyer et al. (2022)used sMRI data (GM, surface area, and local gyrification) in preschoolers to predict literacy ability 2 years later. This study is particularly interesting because children were tested before they were exposed to formal education. This finding lends support to the argument that neuroimaging measures may be used as a way to improve the early detection of learning difficulty, in order to prevent difficulties later on (Mascheretti et al., 2017).

Some longitudinal neuroimaging studies have also attempted to use neural data to classify between children with and without dyslexia. For example,Hoeft et al. (2011)showed that a machine-learning classifier can distinguish whether certain dyslexic children will improve their reading skills or not 2.5 years later using fractional anisotropy (FA) of dMRI and task-fMRI (rhyme judgment) data.Skeide et al. (2016)also reported successful classification of future dyslexia based on sMRI (GM) data in children before formal education. Finally,Yu et al. (2020)demonstrated classification of children with and without familial risk of dyslexia using task-fMRI data (phonological processing) before formal education. These reports suggest that prediction of language ability before formal education may be applicable to the early detection of risk of language deficits. In sum, both cross-sectional and longitudinal designs suggest that neuroimaging data may have the potential to predict literacy skills and classify language disorders.

## 4 Can Neuroimaging Studies Predict Numeracy Skills?

As is the case for studies on literacy, neuroimaging studies that attempt to predict numeracy skills can be categorized as either cross-sectional (Table 3) or longitudinal (Table 4). Cross-sectional studies include, for example,Ullman and Klingberg (2017), who estimated math scores of 6- to 7-year-old children through a prediction model of brain age using dMRI (FA).Pina et al. (2022)predicted four types of math scores (math fluency, calculation, applied problems, quantitative concepts) using 100 radiomics features derived from sMRI data.

**Table 3.** Cross-sectional prediction studies for numeracy.

Study	Target ability/groups	Sample size	Mean age/age range	Data type	Technique	Cross-validation	Max prediction accuracy	Brain areas	Selection method of brain areas
Rykhlevskaia et al. (2009)	Dyscalculia	47 (23 dyscalculia, 24 TD)	Dyscalculia, 8.8; TD, 8.9	dMRI (number of pathways)	SVM	10-fold CV	70.0%	58 ROIs located in the posterior part of the brain	Predetermined ROIs. Independent
Mórocz et al. (2012)	Dyscalculia and dyslexia	58 (36 control, 13 dyscalculia, 9 dyslexia)	TD, 25.6; Dyscalculia, 22.5; dyslexia, 24.6	task-fMRI (multiplication)	Nonlinear SVM	LOOCV	Unclear	24 ROIs across frontal, parietal, temporal, occipital cortices, and cerebellum	Predetermined ROIs. Independent
Dinkel et al. (2013)	Dyscalculia	32 (16 dyscalculia, 16 TD)	Dyscalculia, 8.2; TD, 8.2	task-fMRI (dots comparison and calculation)	Linear SVM	LOOCV	87.5% (dot comparison)	Bilateral IPS, L. thalamus, R. paracentral lobule, R. frontal operculum, R. cingulate gyrus	Predetermined ROIs. Independent
Jolles et al. (2016)	Dyscalculia	38 (19 dyscalculia, 19 TD)	Dyscalculia, 8.9; TD, 8.8	rest-fMRI (FC)	Linear SVM	LOOCV	L. IPS, 84.2%; R. IPS, 76.3%	Bilateral IPS	Predetermined ROIs. Independent
Ullman and Klingberg (2017)	Math and working memory scores	First sample, 82; second sample, 31	First sample, 6-20; second sample, 6.8	dMRI (FA)	Linear SVR	LOOCV	Working memory, *r* = 0.50; math, *r* = 0.41	No specific information	N.A.
Peters et al. (2018)	Dyscalculia and dyslexia	52 (14 dyslexia, 8 dyscalculia, 8 comorbid, 22 TD)	10.8	task-fMRI (subtraction)	Unclear	Repeated LPOCV (Leave-pair-out CV, 1000 times)	Unclear	Frontal, parietal, temporal, and occipital cortices	Predetermined ROIs. Independent
Torres-Ramos et al. (2020)	Math achievement level	57 (18 High, 20 average, 19 low achievements)	High, 8.6-9.9; average, 8.2-9.9; low, 8.3-10.8	task-EEG (digits comparison, FC)	Decision trees	10-fold CV	80.0% (alpha band)	Alpha, beta, delta, theta bands	N.A.
Shim et al. (2021)	Mathematician and nonmathematicians	44 (21 mathematicians, 23 nonmathematicians)	Mathematicians, 33.4; nonmathematicians 27.2	rest-fMRI (FC)	SVM	LOOCV	90.9% (with 39 connection features)	46 pairs of ROIs across the brain	Predetermined ROIs. Nonindependent
Liu et al. (2022)	Math and nonmath students	123 (72 math, 51 nonmathematicians)	Unclear	sMRI	ANN (MLP and ResNet)	5-fold CV	91.8%	L. MFG	Predetermined ROI. Independent
Pina et al. (2022)	Math scores	77	9.7	sMRI (100 radiomics features)	RF regression	Repeated 5-fold CV (20 times)	Unclear	15 regions across frontal and parietal cortices	Prediction accuracy
Ventura-Campos et al. (2022)	Groups with reversal error or not using algebraic problems	20 (10 reversal error, 10 without error)	Reversal error, 21.3; without error, 21.7	task-fMRI (algebra)	13 methods (DA, ANN, SVM, RF, kNN)	LOOCV	80.0% (flexible DA)	8 ROIs across frontal and parietal cortices	Predetermined ROIs. Nonindependent

IPS, intraparietal sulcus.

**Table 4.** Longitudinal prediction studies for numeracy.

Study	Target ability/groups	Sample size	Mean age/age range	Data type	Technique	Cross-validation	Max prediction accuracy	Brain areas	Selection method of brain areas
Supekar et al. (2013)	Improvements in math scores after 8 weeks of training	40 (24 with training, 16 control)	T1/T2: with training, 8.5; control, 9.0	sMRI (GM), rest-fMRI (FC)	Linear regression	4-fold CV	*r* = 0.45	R. hippocampus	Predetermined ROIs. Independent
Qin et al. (2014)	Improvements in the frequency of retrieval-strategy use 1.2 years later	28	T1: 8.3, T2: 9.5	task-fMRI (addition, FC)	Linear regression	4-fold CV	*r* = 0.71	R. hippocampus, L. IPS, bilateral DLPFC	Predetermined ROIs. Nonindependent
Evans et al. (2015)	Math scores up to 6 years later	43	T1: 8.7, T2: unclear	rest-fMRI, sMRI (GM)	Linear SVR	4-fold CV	R ^2^ = 0.44	L. FG, L. IPS, L. DLPFC, L. VLPFC, R. premotor cortex, R. cuneus	Predetermined ROIs. Nonindependent
Iuculano et al. (2015)	Dyscalculia after 8 weeks of training	30 (15 dyscalculia, 15 TD)	T1/T2: Dyscalculia, 8.7; TD, 8.5	task-fMRI (addition)	Linear SVM	LOOCV	Before training, 83.3%; after training, 43.3%	17 ROIs across frontal, parietal, temporal cortices, subcortex, and cerebellum	Predetermined ROIs. Nonindependent
Ullman et al. (2015)	Math and working memory scores after 5 and 7 years	272 (224 preterm infants, 46 control)	T1: 40.3 weeks (gestational age), T2: unclear	sMRI (deformation-based morphometry), dMRI (FA)	SVR	LOOCV	*r* = 0.36 at 5 years	No specific information	N.A.
Michels et al. (2018)	Dyscalculia after 5 weeks training	31 (15 dyscalculia, 16 TD)	T1/T2: 9.5	task-fMRI (number order judgment)	Unclear	LOOCV	Before training, 86.4%; after training, 38.9%	Unclear	Predetermined ROIs. Nonindependent
Schwartz et al. (2020)	Math scores 1.5 years later	31	T1: 11.0, T2: 12.6	task-fMRI (reasoning)	Kernel ridge regression	LOOCV	*r* = 0.39	R. IPS	Predetermined ROIs. Nonindependent
Kuhl et al. (2021)	Dyscalculia	30 (15 dyscalculia, 15 TD)	T1: Dyscalculia, 4.1; TD, 5.0; T2: 7-9	rest-fMRI (ALFF, regional homogeneity, degree centrality), dMRI (streamline density)	SVM	10-fold CV	86.7%	R. IPS, R. DLPFC	Searchlight
Chang et al. (2022)	Improvements in math scores after 4 weeks of training	52 (18 dyscalculia, 34 TD)	T1/T2: 8.2	rest-fMRI (FC)	Linear regression	4-fold CV	*r* = 0.33	Bilateral hippocampus, L. IPS	Predetermined ROIs. Nonindependent

DLPFC, dorsolateral prefrontal cortex; VLPFC, ventrolateral prefrontal cortex.

Other cross-sectional studies have attempted to classify groups of participants with respect to their numeracy skills, for example, those with and without dyscalculia. Dyscalculia is defined as a specific learning difficulty in processing numerical information, learning arithmetic facts, and performing calculations, with otherwise normal intelligence (American Psychiatric Association, 2013). For example,Rykhlevskaia et al. (2009),Jolles et al. (2016), andDinkel et al. (2013)showed that children with and without dyscalculia could be classified using dMRI (number of pathways), rest-fMRI (FC), and task-fMRI data (dots comparison and calculation), respectively. Moreover,Mórocz et al. (2012)andPeters et al. (2018)showed that arithmetic task-fMRI data can be used to classify both dyscalculic and dyslexic children.Torres-Ramos et al. (2020)also showed that task-EEG data (digits comparison) could be used to classify children according to three different categorical levels of math achievement.

Several studies have focused on classifying other aspects of individual differences in numeracy skills.Shim et al. (2021)andLiu et al. (2022)reported classification of individuals based on their expertise in mathematics using rest-fMRI (FC) and sMRI data, respectively.Ventura-Campos et al. (2022)classified individuals who make errors in variable selection (reversal error) when writing equations to given word problems using algebra task-fMRI data.

In contrast to what has been done in studies focusing on literacy, a greater number of studies have used a longitudinal design to predict numeracy skills (Table 4). In a seminal study relying on multivariate regression,Supekar et al. (2013)showed that sMRI (GM) and rest-fMRI (FC) data could predict improvements in math performance of 8-year-old children after 8 weeks of tutoring program consisting of conceptual instruction and speeded arithmetic fact retrieval.Evans et al. (2015)further showed that prediction of longitudinal math outcome is possible even 6 years later using sMRI (GM) and rest-fMRI data.Chang et al. (2022)also reported similar prediction of change in performance after 4 weeks of training using rest-fMRI (FC) data.Schwartz et al. (2020)used fMRI data during a transitive reasoning task to predict math calculation skills 1.5 years later.Ullman et al. (2015)showed that math and working memory scores could be predicted at ages 5 and 7 from neonatal dMRI (FA), but not from sMRI data. Therefore, studies show that numeracy skills may be predicted from brain activity associated with domain-general processing, consistent with the role of these processes in math learning (Raghubar et al., 2010).

We found only one longitudinal neuroimaging study that focused on the classification of dyscalculia as is depicted inFigure 1.Kuhl et al. (2021)classified future dyscalculia at ages of 7-9 years and typically-developing (TD) children based on dMRI and rest-fMRI data before formal education (at ages of 3-6 years). Overall, similar to language abilities, studies show that neuroimaging data may have the potential to predict numeracy skills and classify their disorders.

Note that some longitudinal studies do not neatly fall into the categories described inFigure 1. For example,Qin et al. (2014)used differences between addition task-fMRI data from two time points (T1 and T2, 1.2 years later) to predict improvements in the frequency of retrieval strategy for addition problem solving.Iuculano et al. (2015)showed that task-fMRI data (mental addition) can discriminate between children with and without dyscalculia before (but not after) 8 weeks of a tutoring program involving conceptual instruction and speeded arithmetic fact retrieval training.Michels et al. (2018)also reported similar results based on 5 weeks of mental number line training. These studies represent different ways to combine machine learning with neuroimaging data to explain differences in numeracy skills.

## 5 Can Neuroimaging Studies Predict Other Skills Relevant to Academic Achievement?

In our review of studies above, we exclusively focused on studies that have examined literacy and numeracy skills. However, studies have also tested whether neuroimaging may predict other skills that are relevant to academic achievement. This is notably the case for vocal communication. For example,Abrams et al. (2016)used task-fMRI data from 10-year-old children listening to their mother’s voice to predict children’s communication scores. This is also the case for affective traits related to academic achievement, particularly numeracy skills.Young et al. (2012), for example, classified children with high and low math anxiety groups using task-fMRI (addition and subtraction).Chen et al. (2018)predicted individual differences in positive attitudes toward mathematics using right hippocampal activity during an addition task.Supekar et al. (2015)showed that activity changes in task-fMRI during addition task can predict changes in children’s math anxiety elicited by the same tutoring program. Finally, studies have attempted to use brain information to enhance the diagnosis of autism spectrum disorder (ASD) and attention-deficit/hyperactivity disorder (Eslami et al., 2020;Nogay & Adeli, 2020), both of which can have impact on academic achievement (Arnold et al., 2020;Whitby & Mancil, 2009).Iuculano et al. (2014)notably used task-fMRI data (mental addition) to classify between ASD and TD children, suggesting a potential relationship between the autistic trait and numeracy skills. While these developmental disorders are beyond the scope of this paper, they are important targets that cannot be ignored when considering the overall application of neuroimaging and machine learning to education.

In addition to predicting literacy and numeracy skills, studies have also used brain imaging data to predict academic achievement more generally. For example,Wang et al. (2019)predicted students’ academic achievement at ages 17-20 years using sMRI data.Rasheed et al. (2021)predicted academic achievement (math and language test scores) of school children 4 years later using EEG data.Maglanoc et al. (2020)used a large sample of rest-fMRI data from the UK Biobank to predict educational attainment (based on the qualification variables, e.g., university degree). Studies have also investigated to what extent domain-general skills contributing to academic achievement may be predicted using neuroimaging, including working memory, attention, and intelligence. For example,Ullman et al. (2014)used sMRI and task-fMRI during a visuospatial working memory task to predict children’s working memory capacity 2 years later. There are also a large number of studies on the prediction of intelligence quotient scores from brain data (seeVieira et al. (2022)for a recent systematic review). For example,Greene et al. (2018)used both rest- and task-fMRI data with working memory and emotion identification tasks and found that task-fMRI models outperformed rest-fMRI model in predicting fluid intelligence scores. Therefore, a number of studies provide evidence that neuroimaging may predict general cognitive functioning, though this may not be as relevant as the prediction of specific academic skills such as reading or math for the purpose of identifying children with specific learning difficulties.

## 6 Are there any Specific Brain Circuits Supporting Prediction of Academic Outcomes?

The studies reviewed here are important not only for practical reasons (i.e., to predict outcomes), but also for understanding the brain mechanisms supporting literacy and numeracy acquisition.Tables 1–4report the main brain regions that have been identified in the specific studies.

Some consistency can be seen across studies. For example, studies that have used MRI data to classify participants with and without dyslexia have often identified the left fusiform gyrus (FG) (Skeide et al., 2016;Tamboer et al., 2016;Yu et al., 2022;Zahia et al., 2020), and the left superior temporal gyrus (STG) (Joshi et al., 2023;Płoński et al., 2017;Usman et al., 2021;Zahia et al., 2020) as a potential neuromarker of the condition (see SupplementaryTable S1for a list of studies only focusing on dyslexia). Studies that have used MRI data to classify participants with and without dyscalculia have instead often identified the right intraparietal sulcus (IPS) (Dinkel et al., 2013;Jolles et al., 2016;Kuhl et al., 2021) (see SupplementaryTable S2for a list of studies only focusing on dyscalculia). Although the number of studies remains too limited to quantify the consistency of these findings in a meta-analysis, these findings suggest that these specific brain circuits may be important for academic learning and be the target of future studies.

However, as can also be seen from the tables, the brain systems identified between studies are wide and span the frontal, temporal, parietal, and occipital cortices, as well as subcortical areas. To some extent, this variability is expected given the different domains (e.g., literacy vs., numeracy), brain measures (e.g., EEG, fMRI, sMRI), and tasks (e.g., addition vs. reasoning) explored between studies. Another factor contributing to such variance may be the use of different tests to estimate math and reading scores, and inconsistent definitions of conditions such as dyscalculia and dyslexia. For example, while some studies (e.g.,Jolles et al., 2016) considered children with dyscalculia as having at or below the 25^th^percentile using standardized math test scores, others (e.g.,Dinkel et al., 2013) have used more stringent criteria and focused on children having at or below the 10^th^percentile. In other words, variability in findings is expected given the wide variability in methods between studies. In what follows, we will argue that some critical differences in both machine-learning algorithms and cross-validation methods used between studies might also underlie some of this variability.

## 7 Studies use a Range of Machine-Learning Methods

As shown inFigure 1, neuroimaging studies predicting academic outcomes can be classified as belonging to one of the four categories. However, studies largely differ with respect to the specific machine-learning algorithms they rely on to predict behavior, which is the first important source of variability in the literature. Many classification studies have used linear support vector machine (SVM) (Tables 1–4). Briefly, SVM is a supervised classification algorithm that constructs a set of hyperplanes separating given classes in a high dimensional space, so as to maximize the distance between the nearest data points of any class (Cortes & Vapnik, 1995). The SVM, which is implemented in several decoding toolboxes as a default method (e.g., The Decoding Toolbox;Hebart et al., 2014), is useful for classifying among different groups, such as children with learning disability versus controls. However, studies have also used other techniques, such as logistic regressions (Cui et al., 2016), decision trees (Torres-Ramos et al., 2020), random forest (RF) (Nemmi et al., 2023), naïve Bayes classifiers (NBCs) (Formoso et al., 2021), discriminant analysis (DA) (Bach et al., 2013), k-nearest neighbors (kNN) (Ventura-Campos et al., 2022), and artificial neural networks (ANNs) (Tomaz Da Silva et al., 2021).

A number of different machine-learning methods have also been used in regression studies, though there is more homogeneity among these studies than among classification studies. For instance, some studies have used linear regression, while others have used support vector regression (SVR) (He et al., 2013), relevance vector regression (Yuan et al., 2023), kernel ridge regression (Schwartz et al., 2020), and elastic net (Beyer et al., 2022). Simple or multiple linear regression requires a reduction of input data into a limited number of variables, which has been achieved by focusing on predetermined regions of interest (Hoeft et al., 2007;Supekar et al., 2013) or connectivity among them (Chang et al., 2022). However, inclusion of too many parameters can cause models to overfit the training data that contain non-negligible amount of noise, resulting in reduced generalizability to test data (Bishop, 2006). Elastic net and other regularized regression methods implement constraints on the model weight values to minimize overfitting to the training data and are appropriate for high-dimensional brain data. More recently, connectome-based predictive modeling (CPM) based on linear regression has been adopted for the analysis of brain-behavior association (Shen et al., 2017). For example, researchers have used this technique to analyze the HCP dataset, which includes a large number of subjects (Kristanto et al., 2020;Tomasi & Volkow, 2020).

Although the use of different algorithms is in itself not problematic, it may become so when no justification is given for using one method instead of another. This is unfortunately often the case in the literature. This methodological flexibility increases the researcher degrees of freedom and makes it difficult to parse out exploratory from confirmatory findings, especially given an absence of preregistration across studies (Poldrack et al., 2017). There is also a need for more direct comparison between methodologies. For instance,Płoński et al. (2017)tested SVM, logistic regression, and RF for the same dataset, and reported that logistic regression showed the highest classification accuracy for dyslexia. Furthermore,Ventura-Campos et al. (2022)compared 13 different classification methods and reported that flexible discriminant analysis outperformed other methods. This type of systematic approach can ensure the robustness of results independent of the analysis method. However, this also requires researchers to systematically adopt the most robust methods, which might not always be the case. For example, a meta-analysis on machine-learning application for disease prediction reported that SVM is the most frequently used algorithm in the literature, while RF shows superior accuracy (Uddin et al., 2019). By comparing six regression methods,Cui and Gong (2018)reported that least absolute shrinkage and selection operator (LASSO) regression were worse than the other algorithms when using FC of rest-fMRI data, while ordinary least-square regression was worse when using the sum of FC from each brain region, suggesting that performance of different algorithms also depends on preprocessing methods of the same brain data. To our knowledge, it remains unclear which method is more effective for predicting academic achievement.

Another source of variability in machine-learning methods is the cross-validation (CV) method employed (e.g., split-half, 10-folds, leave-one-out). CV is a widely known method in machine learning to iteratively split some data into training and test samples, testing the model generalizability while minimizing selection bias. In the case of k-fold CV, 1/k of the original data are selected as test samples in each iteration and this procedure covers all original data with k iterations. In contrast, leave-one-out CV (LOOCV) uses each individual data (e.g., subject) as a test sample and iterates across all data. Among the studies included in the current review, LOOCV was the most widely adopted (23 studies), while other studies used various types of k-fold CV methods (10-fold CV, 8 studies; 4-fold CV, 6 studies). Recent studies suggested that the repeated random splits method is more reliable than the leave-one-out method (Valente et al., 2021;Varoquaux et al., 2017). In this method, CV based on different random sample splitting is repeated for multiple times and averaged (e.g., 100 times); 10 studies adopted this technique (Beyer et al., 2022;Nemmi et al., 2023). Overall, there is wide variability in the machine-learning techniques used in neuroimaging studies, in terms of both algorithm selection and CV method. Both of these may have substantial influence on the model performance. This calls for a standardization in the field and future research would require careful consideration of their methodological choices.

## 8 Limitations and Future Directions

As reviewed here, an increasing number of neuroimaging studies suggest that brain data can be used to predict individual differences in both literacy and numeracy skills, as well as other skills relevant for academic achievement. However, several limitations are apparent in the literature.

First, the majority of articles reviewed here have used sMRI or resting fMRI data (Tables 1–4). Although some studies have used task-fMRI data, their sample size was also generally smaller than sMRI and rest-fMRI studies. However, task-fMRI data can contribute to more accurate prediction of individual differences in academic achievement. For example, a recent study has reported superiority of movie-watching task-fMRI data in predicting various cognitive and emotional traits compared with rest-fMRI data (Finn & Bandettini, 2021;Greene et al., 2018). Combining multiple task-fMRI data may further increase prediction performance (Hammer et al., 2015). Moreover, task-fMRI can shed light on the heterogeneous profiles of children with dyscalculia or dyslexia, who might have specific difficulties in some cognitive skills (such as phonological or visual attentional deficits in the case of dyslexia) by targeting appropriate ROIs (Jednoróg et al., 2014;van Ermingen-Marbach et al., 2013).

Second, the literature is largely dominated by MRI data and relatively few studies have used EEG, MEG, or fNIRS in predictive studies. For instance, to the best of our knowledge,Dimitriadis et al. (2018)was the only example of using MEG data to predict language disorders.Lei et al. (2020)was also the only example of using fNIRS data to predict second language proficiency. The wide usage of MRI data might be due to its advantage in spatial resolution compared with the other methods. Considering their portability, however, EEG, fNIRS, and optically pumped magnetometers (OPM)-MEG (Boto et al., 2018;Brookes et al., 2022), as well as portable MRI (Liu et al., 2021), are interesting because they are more accessible for experimentation in schools and clinical practices than conventional MRI (Stangl et al., 2023). Given that early detection of potential learning disabilities is an important goal of several neuroimaging studies discussed here (Hoeft et al., 2007), efforts should be made to evaluate the potential of task-related portable neuroimaging data for predicting outcomes in children.

Third, most previous studies recruited subjects who were already exposed to formal education. However, predicting outcomes from neuroimaging data may be most interesting before potential difficulties occur at the behavioral level. That is, brain data might help detect a risk for learning disabilities before children begin formal education, which may help ensure that children receive appropriate educational support at the earliest stage. To our knowledge, four studies in the literacy domain (Beyer et al., 2022;Skeide et al., 2016;Yu et al., 2020;Zare et al., 2016) and two studies in the numeracy domain (Kuhl et al., 2021;Ullman et al., 2015) tested children before the onset of formal education. Most of these studies used either sMRI or rest-fMRI, and only one study used task-fMRI data (Yu et al., 2020). The relative lack of studies might reflect the inherent difficulty of pediatric MRI with young children. Again, this calls for the use of more child-friendly portable measurement techniques to inform about the prediction of future academic outcomes.

Fourth, there is still room for the integration of sophisticated machine-learning methods. Although linear regression and SVM are the two most widely used techniques in previous studies, some recent studies have adopted ANNs (Joshi et al., 2023;Tomaz Da Silva et al., 2021;Zahia et al., 2020). ANN is a computational model inspired by biological neural networks (BNNs). It consists of multiple layers of neuronal units, where the weighted sum of units in one layer is used as input for the next layer after a nonlinear transformation. One advantage of using ANNs is that one can compare commonality between ANNs and BNNs in terms of their representations across different layers/regions (Goldstein et al., 2022;Nakai & Nishimoto, 2023;Schrimpf et al., 2021). However, it remains unclear which ANN model is the more appropriate to explain developmental changes in brain representations and differences between those with and those without learning disabilities. Cross-validation techniques might also be improved. Although the large majority of studies use left-out sample predictions, this method is not the only method for brain-based classification or regression.Siegelman et al. (2021), for example, recently proposed a Bayesian latent-mixture model framework to classify between children with and without dyslexia. This framework does not need left-out samples because it constructs classification models by only using neuroimaging data without any categorical labels. In other words, it interprets the fit between the models’ classification and categorical labels as an estimate of its explanatory power. On the other hand,Astle et al. (2019)used unsupervised self-organization map to classify children into four groups (typically developing, broad cognitive deficits in both language and mathematics, working memory problems, and phonological difficulties). These alternative approaches can shed light on the search for more effective methods for predicting academic achievement.

Fifth, a critical step for any neuroimaging studies using machine learning is feature selection. As is clear from our survey of the literature, many studies have relied on the selection of specific regions-of-interest (ROIs) as features to construct machine-learning models (seeTables 1–4). A well-known issue with ROI analyses in neuroimaging studies is that the way they are selected might bias the outcome of the analyses. For instance, selecting ROIs based on data that are nonindependent from the effect tested might lead to effect sizes that are inflated, an issue known as circular analyses (Kriegeskorte et al., 2009). Several neuroimaging studies (i.e., 15 out of 30 ROI-based studies) reviewed here have selected ROIs based on the same dataset that was used for their machine-learning analyses. This may cause inflation of decoding accuracy and result in a lack of generalizability of decoding models, even if ROIs are selected using univariate analyses and subsequently tested with multivariate analyses. The use of nonindependent ROIs may further be inconsistent with the assumption of the left-out sample prediction because the test samples are already used for the feature selection during model training. Therefore, studies using nonindependent ROIs could be considered as confirmatory, much like those that use in-sample correlations between two datasets (Dumontheil & Klingberg, 2012). Other feature selection methods may be used to circumvent this circularity issue. For example, some have interpreted contributing voxels based on nonzero decoding model weight values (Cui et al., 2018;Hoeft et al., 2011) or based on the nested cross-validation (Cui et al., 2016). Although caution is needed in interpreting weight values (Haufe et al., 2014), both approaches can minimize bias of contributing brain regions. We believe that an interesting approach to avoid circularity issues in feature selection is searchlight decoding analysis (He et al., 2013;Kuhl et al., 2021). This whole-brain analysis constructs decoding model using voxels included in spheres centered around each cortical voxel. This makes it possible to identify brain regions in which multivoxel patterns are sensitive to the difference between conditions or subject groups (Kriegeskorte et al., 2006).

Sixth, because we attempted to provide a comprehensive review of the literature, several studies discussed here rely on relatively small sample sizes (seeTables 1–4). It is now acknowledged that small sample sizes can lead to a significant lack of reliability in neuroimaging data (Button et al., 2013). Therefore, conclusions from these studies must be considered with caution. Indeed, prediction accuracy can largely vary based on sample size. For example,Tamboer et al. (2016)classified dyslexia with 80.0% accuracy in a relatively small group of participants (N = 49), while they obtained 59.0% accuracy in a second group with a much larger sample size (N = 876). In the case of classification between learning disability (dyslexia or dyscalculia) and typically developing participants, no study with a large sample size (N > 100) achieved more than 80% accuracy (SupplementaryTables S1 and S2).Usman et al. (2021)did report 94.7% accuracy with N = 148, but this study classified MRI image patches and did not directly classify original brain data. Overall, this suggests that a machine-learning model with a classification accuracy of 80%, even if the accuracy is significantly higher than the chance level, would lead to misdiagnosis in one subject out of five. This is relatively low for real-world applications, which should aim for highly accurate predictions more than statistical significance.

Finally, as is the case generally in neuroimaging research, openly sharing data will be fundamental to improve models predicting academic outcomes from brain data. Building reliable predictive models requires a large amount of data (Varoquaux, 2018). Eight studies constructed predictive models of literacy skills (Table 1) using such open datasets. In addition to the neuroimaging data published in the Adolescent Brain Cognitive Development (ABCD) study (Casey et al., 2018) or in UK Biobank (Littlejohns et al., 2020), researchers have published a series of open task-fMRI datasets of school children (Lytle et al., 2019,^2020^;Suárez-Pellicioni et al., 2019;Wang et al., 2022). Such large neuroimaging datasets will be beneficial for future developments in predicting academic performance using machine learning. In addition, acceleration of open data and codes would enable comparison of prediction accuracy across different studies and may reduce inconsistencies between studies.

## 9 Are we Getting Closer to Real-World Applicability?

In their review,Gabrieli et al. (2015)highlighted a number of challenges that would have to be met by neuroimaging studies predicting skills to have some real-world applicability, either in the classroom or in a clinical context. These notably included the reliability and representativeness of the findings, the added value compared with behavioral indicators, the economic cost, as well as the ethical and societal issues these methods may raise. We revisit here these challenges 9 years afterGabrieli et al. (2015).

The section above already fleshes out the critical limitations and challenges in the body of literature. On the one hand, the relative lack of consistency in methodology, experimental designs, and findings shows that there is much room for improvement for studies aiming to translate their findings to the real world. On the other hand, the literature has significantly expanded over the past 10 years. Although initial studies largely focused on literacy skills, investigation of academic skills has now largely expanded to numeracy. In comparison with earlier ones, studies have also now started to focus on long-term outcomes, sometimes over the course of several years (e.g., seeKuhl et al. (2021)for long-term prediction of dyscalculia). This is critical if neuroimaging is to be thought about as a tool for enhancing the detection of future learning difficulties before they occur (Raschle et al., 2012). Finally, recent technical advances in machine learning, as well as the availability of large-scale neuroimaging data, might accelerate practical applications. For example, ANNs with a large number of layers were not available 25 years ago (Liu et al., 2022). The development of machine-learning toolboxes such as scikit-learn (Abraham et al., 2014) has also reduced the barriers to attempting prediction analyses using neuroimaging data.

For neuroimaging measures to be useful indicators for clinical practice or in the classroom, they would of course need to add some explanatory power to the prediction of future academic skills that can already be gathered from behavioral assessments alone. Some studies suggest that a combination of behavioral and brain-based measures may outperform either behavioral or neuroimaging measures alone when predicting academic skills (Beyer et al., 2022;Hoeft et al., 2007), though most studies still lack a systematic comparison of prediction based on neuroimaging and behavior.

Most studies reviewed here have used MRI to predict academic achievement. Some common criticisms of MRI include its cost and accessibility, as well as the fact that pediatric MRI is relatively challenging. As also pointed out byGabrieli et al. (2015), it would be important for any financial analysis to account for current practices, which may be costly and less effective as they are often targeted at children who are already failing school. Even though MRI may not be used in the population at large, some studies do suggest that early MRI measures may be useful for some targeted population, for example, for children of parents with learning disabilities. Indeed, a large body of evidence indicates that such children are at greater risk of developing the disability than their peers. Brain-based measures, together with behavioral assessments, may thus enhance the early detection of at-risk children (Beyer et al., 2022;Kuhl et al., 2021). Another path for reducing the economic cost associated with collecting brain-based measures is a greater reliance on portable and wearable neuroimaging devices, such as wireless EEG or fNIRS. Critically, these methods have been increasingly used over the past 10 years, with several studies showing their applicability for collecting brain data in uncontrolled environments such as classrooms (Davidesco et al., 2021). The field is now ripe for testing how these techniques may be combined with machine learning to predict academic outcomes and how they compare with MRI measures.

Finally, any use of neuroimaging measures to predict aspects of academic achievement would have to take into consideration ethical and societal issues. Though behavioral measures such as intelligence quotient (IQ) have long been used to predict academic achievement (Chamorro-Premuzic & Furnham, 2008), studies have shown that brain-based measures may have a special status in the public eye and be easily misinterpreted (Racine et al., 2005). For example, there is evidence suggesting that people often perceive scientific claims as more credible when they include references to the brain or neuroscientific information (Weisberg et al., 2008), which suggests that people might give more weight to brain-based than behavioral indicators. Another critical aspect of the findings reviewed here is that they may raise ethical questions about whether they could be used to merely identify those with the highest likelihood of success instead of identifying individuals who are at risk and would need help. Although a discussion of these ethical and societal issues is beyond the scope of the present review, it is clear that they need to be considered by researchers, clinicians, educators, parents, students, and policy makers.

## 10 Conclusion

Nine years after the review ofGabrieli et al. (2015), studies using machine learning to predict educational achievement and learning disabilities from brain activity have grown exponentially, particularly in the domains of literacy and numeracy. However, we found in this updated review a considerable variation in algorithms and underlying brain circuits between studies. Studies also largely rely on relatively small samples and suboptimal models. We argue that the field needs a standardization of methods, as well as a greater use of accessible and portable neuroimaging methods that have more applicability potential than lab-based neuroimaging techniques.

## Supplementary Material

Supplementary Material

## Data Availability

There are no data or code associated with this article.
