# Supplementary material for: From brain to education through machine learning: Predicting literacy and numeracy skills from neuroimaging data
Source: Imaging Neurosci (Camb). 2024 Jul 3;2:imag-2-00219. doi: 10.1162/imag_a_00219 (PMC12272187; doi:10.1162/imag_a_00219)
Supplement: Supplementary Material [file imag_a_00219-supp.pdf]

Supplementary Information

Inclusion criteria of the reviewed articles

Our literature search started by including all articles regarding literacy and numeracy referred in the review by (Gabrieli et al., 2015). We then performed an additional literature search on PubMed (https://pubmed.ncbi.nlm.nih.gov/) using (1) neuroscience terms ("brain" OR “neuro*”), (2) ability terms (“learning” OR “skill*” OR “score*” OR “abilit*” OR “achievement*” OR “disabilit*” OR “deficit*”), (3) machine-learning terms (“machine learning” OR “classifier*” OR “decoder*” OR “multivariate” OR “searchlight”[tiab] OR “cross validation”). These three term sets were concatenated together using AND connectives, and were further concatenated with (4) either literacy (“literacy” OR “language*” OR “reading” OR “linguistic*” OR “dyslexia”) or numeracy terms (“numeracy” OR “math*” OR “arithmetic*” OR “dyscalculia”) using an AND connective. All terms were specified with the [tiab] code to search within the title and abstract of the articles. The literature search was conducted on May 3rd, 2023. We found 1,136 results for literacy and 321 results for numeracy.

From these results, we excluded studies with non-human subjects or patients (e.g., Alzheimer disease), review or meta-analysis, those primarily focusing on methodological development, and those related to other cognitive factors (e.g., emotion recognition). Because this literature search might still miss several articles that do not contain key terms in the title of abstract, we also manually added articles that were found based on the citation relation from those found in the literature search. We further excluded studies that did not use machine learning methods in the way assumed in the current review (i.e., across subjects, using left-out samples prediction). Accordingly, 34 articles were included in the literacy domain, and 20 articles were included in the numeracy domain.

Information from all articles were independently coded by two raters (first author, T.N., and second author, C.T.). Tables 1-4 were generated by integrating coding results by the two raters. All cases of inconsistent coding were solved by having the two raters come to an agreement, and their interpretation was confirmed by the last author (J.P.).

Table S1. Studies on dyslexia classification

Study	Target ability/groups	Sample size	Mean age/age range	Data type	Technique	Cross-validation	Max prediction accuracy	Brain areas	Selection method of brain areas
Tamboer et al. (2016)	Dyslexia	First sample: 49 (22 dyslexia, 27 TD); second sample: 876 (60 dyslexia, 816 TD)	First sample, dyslexia, 20.7; TD, 20.3; second sample, dyslexia, 22.5; TD, 22.9	sMRI (GM)	Linear SVM	LOOCV	First sample, 80.0%; Second sample, 59.0%	L. IPL, bilateral FG	Predetermined ROIs. Independent
Cui et al. (2016)	Dyslexia	61 (28 dyslexia, 33 TD)	Dyslexia, 11.6; TD, 11.8	sMRI (WM), dMRI (FA, mean, axial, radial diffusivity)	Linear SVM, Logistic regression	LOOCV	83.6% (SVM)	43 (SVM) and 40 (Logistic regression) connections across the brain	CV within training data
Płoński et al. (2017)	Dyslexia	236 (130 dyslexia, 106 TD)	8.5-13.7	sMRI (volume, cortical thickness, surface area, folding index, and mean curvature)	Linear SVM, Logistic regression, RF	LOOCV and repeated 10-fold CV (100 times)	65.0%	L. MTG, L. STG, L. frontal pole, L. precuneus	CV within training data
Cignetti et al. (2020)	Dyslexia, DCD	136 (45 dyslexia, 20 DCD, 29 comorbid, 42 TD)	Dyslexia, 10.2; DCD, 10.0; comorbid, 10.2; TD, 10.1	rest-fMRI (FC)	Linear SVM	LOOCV	75.9% (comorbid vs. TD)	Default mode, dorsal attention, ventral attention, frontoparietal networks	Weight values
Zahia et al. (2020)	Dyslexia	55 (19 dyslexia, 17 monocular vision, 19 TD)	Dyslexia, 10.5; monocular vision, 10.4; TD, 10.0	task-fMRI (lexical decision, orthographic matching, semantic categorization)	ANN (3D CNN)	4-fold CV	72.3%	Bilateral IFG, MTG, STG, precuneus, FG, L. AG, L. medial temporal	Predetermined ROIs. Independent
Mascheretti et al. (2021)	Dyslexia	44 (22 dyslexia, 22 TD)	Dyslexia, 14.1; TD, 13.2	task-fMRI (visual detection)	Multiple kernel learning SVM	10-fold CV	65.9%	11 ROIs including R. SPL, L. IPL, R. IFG, and occipital cortex	Weight values
Tomaz Da Silva et al. (2021)	Dyslexia	32 (16 dyslexia, 16 TD)	Dyslexia, 9.6; TD, 8.4	task-fMRI (word reading)	four ANNs (grammar-based genetic programming [GGP] CNN, GGP 3D CNN, LeNet-5, LeNet-5 3D), linear SVM	80% for training, 10% for validation, 10% for testing	94.8% (GGP 2D CCN)	Large portions of the frontal, parietal, temporal and occipital cortices	Weight values
Usman et al. (2021)	Dyslexia	Dyslexia, 91; TD, 57	Dyslexia, 11.4; TD, 19-30	sMRI (GM), task-fMRI (rhyming, spelling, semantic decision), rest-fMRI, dMRI	ANN (two-ways cascaded CNN, ResNet-50, Inception V3)	Repeated 10-fold CV (10 times)	94.7% (ResNet50)	L. STG, L. OTG, lateral cerebellum	Predetermined ROIs. Independent
Yu et al. (2022)	Familial risk of dyslexia	98 (35 with familial risk, 63 without risk)	Risk, 8.9; without risk, 8.3	rest-fMRI (FC)	Linear SVM	LPOCV	55.0%	L. FG	Predetermined ROIs. Non-independent. Weight values

Joshi et al. (2023)	Dyslexia	192 (96 dyslexia, 96 TD)	Dyslexia, 9.9; TD, 9.8	sMRI (GM, WM)	ANN (autoencoder), SVM, RF	Repeated sampling (100 times) with 80% for training, 20% for testing	75.0% (ANN)	L. IPL, R. orbitofrontal, L. STG	Classification accuracy with image perturbation
Nemmi et al. (2023)	Dyslexia, DCD	136 (45 dyslexia, 20 DCD, 29 comorbid, 42 TD)	Dyslexia, 10.2; DCD, 1.0; comorbid, 10.2; TD, 10.1	sMRI (GM, WM), rest-fMRI (ALFF, local and global correlation)	RF, linear SVM	Repeated 10-fold CV (10 times)	Dyslexia, 79.0%; DCD, 58.0%; comorbid, 62.0% (SVM)	12 ROIs including L. cerebellum, R. MFG, R. SFG, R. LOC, L. insula, R. putamen, R. insula, and R. STG	Predetermined ROIs. Non-independent
Skeide et al. (2016)	Dyslexia after 1.7 years and at the end of the first grade	First sample: 34 (17 dyslexia, 17 TD); second sample 20 (10 dyslexia, 10 TD)	T1: First sample, dyslexia, 10.4; TD, 10.6; second sample, dyslexia, 5.6; TD, 5.8; T2: unclear	sMRI (GM, WM)	Linear SVM	10-fold CV	First sample: 73.5%; second sample: 75.0%	L. FG	Prediction accuracy, predetermined ROIs. Non-independent
Yu et al. (2020)	Familial risk of dyslexia	81 (35 with risk, 34 without risk, 12 dyslexia and with familial risk)	T1: with risk, 5.5; without risk, 5.4; dyslexia, 5.8; T2: with risk, 8.7; without risk, 9.0; dyslexia, 8.3	task-fMRI (phonological processing)	Linear SVM	15-fold CV	68.3%	R. IFG, L. AG	Searchlight

Table S2. Studies on dyscalculia classification

Study	Target ability/groups	Sample size	Mean age/age range	Data type	Technique	Cross-validation	Max prediction accuracy	Brain areas	Selection method of brain areas
Rykhlevskaia et al. (2009)	Dyscalculia	47 (23 dyscalculia, 24 TD)	Dyscalculia, 8.8; TD, 8.9	dMRI (number of pathways)	SVM	10-fold CV	70.0%	58 ROIs located in the posterior part of the brain	Predetermined ROIs. Independent
Mórocz et al. (2012)	Dyscalculia and dyslexia	58 (36 control, 13 dyscalculia, 9 dyslexia)	TD, 25.6; Dyscalculia, 22.5; dyslexia, 24.6	task-fMRI (multiplication)	Nonlinear SVM	LOOCV	Unclear	24 ROIs across frontal, parietal, temporal, occipital cortices, and cerebellum	Predetermined ROIs. Independent
Dinkel et al. (2013)	Dyscalculia	32 (16 dyscalculia, 16 TD)	Dyscalculia, 8.2; TD, 8.2	task-fMRI (dots comparison and calculation)	Linear SVM	LOOCV	87.5% (dot comparison)	Bilateral IPS, L. thalamus, R. paracentral lobule, R. frontal operculum, R. cingulate gyrus	Predetermined ROIs. Independent
Jolles et al. (2016)	Dyscalculia	38 (19 dyscalculia, 19 TD)	Dyscalculia, 8.9; TD, 8.8	rest-fMRI (FC)	Linear SVM	LOOCV	L. IPS, 84.2%; R. IPS, 76.3%	Bilateral IPS	Predetermined ROIs. Independent
Peters et al. (2018)	Dyscalculia and dyslexia	52 (14 dyslexia, 8 dyscalculia, 8 comorbid, 22 TD)	10.8	task-fMRI (subtraction)	Unclear	Repeated LPOCV (1000 times)	Unclear	Frontal, parietal, temporal, and occipital cortices	Predetermined ROIs. Independent
Iuculano et al. (2015)	Dyscalculia after 8 weeks of training	30 (15 dyscalculia, 15 TD)	T1/T2: Dyscalculia, 8.7; TD, 8.5	task-fMRI (addition)	Linear SVM	LOOCV	Before training, 83.3%; after training, 43.3%	17 ROIs across frontal, parietal, temporal cortices, subcortex, and cerebellum	Predetermined ROIs. Non-independent
Michels et al. (2018)	Dyscalculia after 5 weeks training	31 (15 dyscalculia, 16 TD)	T1/T2: 9.5	task-fMRI (number order judgment)	Unclear	LOOCV	Before training, 86.4%; after training, 38.9%	Unclear	Predetermined ROIs. Non-independent
Kuhl et al. (2021)	Dyscalculia	30 (15 dyscalculia, 15 TD)	T1: Dyscalculia, 4.1; TD, 5.0; T2: 7-9	rest-fMRI (ALFF, regional homogeneity, degree centrality), dMRI (streamline density)	SVM	10-fold CV	86.7%	R. IPS, R. DLPFC	Searchlight. Independent
